# Radiolabeled Monoclonal Antibody Against Colony-Stimulating Factor 1 Receptor Specifically Distributes to the Spleen and Liver in Immunocompetent Mice

**DOI:** 10.3389/fonc.2021.786191

**Published:** 2021-12-16

**Authors:** Stijn J. H. Waaijer, Frans V. Suurs, Cheei-Sing Hau, Kim Vrijland, Karin E. de Visser, Derk Jan A. de Groot, Elisabeth G. E. de Vries, Marjolijn N. Lub-de Hooge, Carolina P. Schröder

**Affiliations:** ^1^ Department of Medical Oncology, University Medical Center Groningen, University of Groningen, Groningen, Netherlands; ^2^ Division of Tumor Biology & Immunology, Oncode Institute, Netherlands Cancer Institute, Amsterdam, Netherlands; ^3^ Department of Immunohematology and Blood Transfusion, Leiden University Medical Center, Leiden, Netherlands; ^4^ Department of Clinical Pharmacy and Pharmacology, University Medical Center Groningen, University of Groningen, Groningen, Netherlands; ^5^ Department of Nuclear Medicine and Molecular Imaging, University Medical Center Groningen, University of Groningen, Groningen, Netherlands

**Keywords:** positron emission tomography (PET), antibody immunotherapy, noninvasive imaging in animal models, pharmacokinetics/pharmacodynamics (PKPD), tumor-associated macrophage (TAM)

## Abstract

Macrophages can promote tumor development. Preclinically, targeting macrophages by colony-stimulating factor 1 (CSF1)/CSF1 receptor (CSF1R) monoclonal antibodies (mAbs) enhances conventional therapeutics in combination treatments. The physiological distribution and tumor uptake of CSF1R mAbs are unknown. Therefore, we radiolabeled a murine CSF1R mAb and preclinically visualized its biodistribution by PET. CSF1R mAb was conjugated to *N*-succinyl-desferrioxamine (*N*-suc-DFO) and subsequently radiolabeled with zirconium-89 (^89^Zr). Optimal protein antibody dose was first determined in non-tumor-bearing mice to assess physiological distribution. Next, biodistribution of optimal protein dose and ^89^Zr-labeled isotype control was compared with PET and *ex vivo* biodistribution after 24 and 72 h in mammary tumor-bearing mice. Tissue autoradiography and immunohistochemistry determined radioactivity distribution and tissue macrophage presence, respectively. [^89^Zr]Zr-DFO-*N*-suc-CSF1R-mAb optimal protein dose was 10 mg/kg, with blood pool levels of 10 ± 2% injected dose per gram tissue (ID/g) and spleen and liver uptake of 17 ± 4 and 11 ± 4%ID/g at 72 h. In contrast, 0.4 mg/kg of [^89^Zr]Zr-DFO-*N*-suc-CSF1R mAb was eliminated from circulation within 24 h; spleen and liver uptake was 126 ± 44% and 34 ± 7%ID/g, respectively. Tumor-bearing mice showed higher uptake of [^89^Zr]Zr-DFO-*N*-suc-CSF1R-mAb in the liver, lymphoid tissues, duodenum, and ileum, but not in the tumor than did ^89^Zr-labeled control at 72 h. Immunohistochemistry and autoradiography showed that ^89^Zr was localized to macrophages within lymphoid tissues. Following [^89^Zr]Zr-DFO-*N*-suc-CSF1R-mAb administration, tumor macrophages were almost absent, whereas isotype-group tumors contained over 500 cells/mm^2^. We hypothesize that intratumoral macrophage depletion by [^89^Zr]Zr-DFO-*N*-suc-CSF1R-mAb precluded tumor uptake higher than ^89^Zr-labeled control. Translation of molecular imaging of macrophage-targeting therapeutics to humans may support macrophage-directed therapeutic development.

## Introduction

The tumor microenvironment comprises several cell types, including fibroblasts and many different immune cells. Recently, macrophages gained attention as an important part of the intratumoral immune cell compartment. A high number of tumor-associated macrophages (TAMs) correlate with a higher tumor grade and a worse prognosis for patients with several cancer types (1–4).

In various preclinical mouse tumor models, TAMs have been targeted with small molecules or antibodies, resulting in depletion, repolarization, activation, or inhibition of recruitment to the tumor (5–12). These strategies synergized with antitumor effects of cancer therapies, including immune checkpoint inhibitors, chemotherapy, and radiotherapy (5–14). Especially in preclinical models of mammary tumors, strong synergistic antitumor effects were seen when treatment modalities were combined with targeting TAMs (9–11, 13, 14). One of these preclinical models is the *K14cre;Cdh1^F/F^;Trp53^F/F^* (KEP) mouse model for spontaneous mammary tumorigenesis. Mammary tumors arising in KEP mice resemble human invasive lobular carcinomas (15) and are strongly infiltrated with macrophages (11). Targeting TAMs by inhibiting their survival in the KEP model provoked a type 1 interferon response, which enhanced the efficacy of platinum-based chemotherapies (11).

One of the major routes for targeting TAMs is inhibition of the colony-stimulating factor-1 (CSF1; CD115)–CSF1 receptor (CSF1R) axis. The CSF1–CSF1R axis is a crucial macrophage survival and differentiation pathway (16). Multiple monoclonal antibodies (mAbs) targeting CSF1R have been developed to disrupt the immunosuppressive tumor microenvironment and are evaluated in early clinical trials. They generally appear tolerable, but monotherapy efficacy is still limited (1, 17). Macrophage targeting as an adjunct to potentiate chemo-, immuno-, or radiotherapy may be more successful. However, insight into the whole-body distribution and tumor uptake of CSF1R mAbs is lacking.

Breast cancer is thought to be relatively insensitive to immunotherapy than other tumor types, which is why combination strategies to improve efficacy are potentially relevant (18). Non-invasive imaging of CSF1R mAb biodistribution could provide information regarding physiological distribution and tumor targeting and thereby support the rational design of combination strategies that include macrophage targeting for breast cancer (19–21). Choosing a preclinical model reflecting the complexity of the tumor immune microenvironment and its components is thereby essential to mimic the human setting.

Therefore, in the present study, we studied the biodistribution of a radiolabeled CSF1R mAb that targets murine CSF1R. To enable radiolabeling, we conjugated the CSF1R mAb with *N*-succinyl (*N*-suc) desferrioxamine (DFO) followed by coupling to PET isotope zirconium-89 (^89^Zr). Thus, its behavior in a mouse model bearing an orthotopically transplanted KEP tumor can be studied. We tested the impact of protein dose on [^89^Zr]Zr-DFO-*N*-suc-CSF1R-mAb pharmacokinetics. Furthermore, with complementary *ex vivo* techniques including autoradiography and immunohistochemistry, mAb localization and the presence of tissue macrophages were assessed.

## Materials and Methods

### Antibody Conjugation and Labeling

Anti-mouse CSF1R mAb (rat IgG2a; clone AFS98) and rat IgG2a isotype control (clone 2A3) were obtained from BioXCell. Antibodies were conjugated with tetrafluorophenol-*N*-succinyl-DFO B-Fe (TFP-*N*-suc-DFO-Fe; ABX). To improve conjugation efficiency, antibodies were concentrated using Vivaspin 2, 10-kDa centrifugal concentrators (Sartorius). The pH was adjusted to 9.5 using 0.1 M of Na_2_CO_3_, followed by a sevenfold molar excess of TFP-*N*-suc-DFO-Fe. After a 1-h incubation at room temperature with mild agitation, conjugation efficiency was determined using a Waters size-exclusion high-performance liquid chromatography (SE HPLC) system. This SE HPLC system is equipped with a dual-wavelength absorbance detector (280 versus 430 nm), and TSK-gel SW column G3000SWXL 5 µm, 7.8 mm (Joint Analytical Systems) using phosphate-buffered saline (PBS; 9.0 mM of sodium phosphate, 1.3 mM of potassium phosphate, 140 mM of sodium chloride, pH 7.4; UMCG; flow 0.7 ml/min) as mobile phase. On average, four molecules of TFP-*N*-suc-DFO-Fe were conjugated to one antibody molecule CSF1R mAb or IgG2a. Next, pH was adjusted to 4.5 using 0.25 M of H_2_SO_4_, and a 50-fold molar excess EDTA was added to remove Fe during 90 min of incubation at 37°C with mild agitation. The reaction mixture was purified using a PD Minitrap G-25 (GE Healthcare) according to manufacturer’s gravity protocol to deplete unbound TFP-*N*-suc-DFO and EDTA. After purification, protein concentration and purity were assessed by UV-Vis spectrophotometry (Cary 60 Agilent) and SE HPLC, respectively.

Thus, obtained purified intermediates DFO-*N*-suc-CSF1R-mAb and DFO-*N*-suc-IgG2a were radiolabeled with [^89^Zr]Zr-oxalate (Perkin Elmer) as described before (22). Radiochemical purity was assessed by a trichloroacetic acid precipitation assay (23), and antibody purity was assessed by SE HPLC using an absorbance detector (280 nm) and in-line radioactivity detector (23).

### CSF1R Binding Assay

Maintained immunoreactivity of DFO-*N*-suc-CSF1R-mAb to CSF1R extracellular domain was determined using an ELISA. A Nunc 96-well polystyrene conical bottom microwell plate (Thermo Fisher Scientific) was coated overnight at 4°C with 1 µg/ml of recombinant mouse CSF1R protein (Sino Biological) in a 100 µl of 0.05 M Na_2_CO_3_ solution, pH 9.6. Next, wells were washed three times with 0.05% polysorbate 20/PBS. The aspecific binding was blocked with a 0.5% bovine serum albumin (BSA; Sigma-Aldrich)/0.05% polysorbate 20/PBS for 2 h. Subsequently, the plate was incubated at room temperature with 100 µl concentration series of parental CSF1R mAb or DFO-*N*-suc-CSF1R-mAb ranging from 0.001 to 20 nM. After 1-h incubation, wells were washed three times and incubated with peroxidase-conjugated rabbit anti-rat polyclonal Ab (P0450; Dako) for 30 min at room temperature. Finally, wells were washed three times and incubated for 5 min with 3,3′,5,5′–tetramethylbenzidine (SureBlue Reserve; Seracare) followed by 1 M of hydrochloric acid to stop the reaction. Absorbance was measured at 450 nm in a microplate reader.

### Animal Experiments

All animal experiments were approved by both the Institutional Animal Care and Use Committee of the University of Groningen and the Netherlands Cancer Institute. Food and water were provided *ad libitum*. Female FVB/N mice of 10–12 weeks of age (Janvier Labs) were studied. Mammary tumors from the KEP mouse model for spontaneous mammary tumorigenesis were collected to be implanted in FVB/N female mice (11, 15). In short, KEP tumors were collected in ice-cold PBS, cut into small pieces, and resuspended in DMEM F12 containing 60% fetal calf serum (FCS) and 20% dimethyl sulfoxide and stored at −150°C. KEP tumor pieces (1 × 1 mm) were placed into the mammary fat pad of FVB/N female mice. Tumor growth was monitored twice weekly by palpation and caliper measurements. The radiolabeled antibody was retro-orbitally injected when tumors reached a size of 200 to 400 mm^3^. Mice were allocated randomly to antibody groups. Antibody doses comprised 0.4 mg/kg (10 µg, 0.067 nmol) of ^89^Zr-labeled antibody, and at higher doses, an unlabeled unconjugated antibody of up to 4 mg/kg (100 µg, 0.67 nmol) or 10 mg/kg (250 µg, 1.67 nmol) was added. Mice were anesthetized during microPET scanning with isoflurane/oxygen inhalation (5% induction, 2.5% maintenance). Details regarding antibody dose, number of animals, microPET scans, and time of biodistribution are included in the figure legends.

### MicroPET Scanning and *Ex Vivo* Biodistribution

All microPET scans were executed in a Focus 200 rodent scanner (CTI Siemens). Mice were kept warm on heating mats. A transmission scan of 515 s was obtained using a ^57^Co point source for tissue attenuation. The reconstruction of microPET scans was performed as previously described (24). After reconstruction, images were interpolated with trilinear interpolation using PMOD software (version 3.7, PMOD Technologies LLC). Coronal microPET images or maximal intensity projection images were used for display. Volumes of interest (VOIs) of the whole tumor were drawn based on biodistribution tumor weight. For the heart, a 92-mm^3^ ellipsoid VOI in the coronal plane was drawn. Furthermore, representative VOIs were drawn for the spleen and liver and subsequently quantified. Data are expressed as the mean standardized uptake value (SUV_mean_).

For all *ex vivo* biodistribution studies, the tumor, whole blood, and organs of interest were retrieved and weighed. Whole blood was collected in sodium heparin tubes (BD) and was fractionated by centrifugal force to obtain plasma. Samples, together with radiolabeled antibody standards, were counted in a calibrated well-type g-counter (LKB Instruments). Antibody uptake is expressed as the percentage injected dose per gram of tissue (%ID/g).

### *Ex Vivo* Autoradiography and Immunohistochemistry

Organs of interest, including tumors, were fixed in formalin (4% paraformaldehyde/PBS) overnight, followed by paraffin embedding. Sections measuring 4 µm were subsequently exposed overnight to a phosphor screen (PerkinElmer) in an X-ray cassette. Signal was detected with a Cyclone Storage Phosphor System (PerkinElmer). Slides used for *ex vivo* autoradiography were deparaffinized. After that, they were stained with H&E and digitalized with NanoZoomer and NDP software (Hamamatsu). Subsequent slides were stained for murine pan-macrophage marker F4/80 with a rat anti-mouse F4/80 mAb (CI:A3; Bio-Rad) by immunohistochemistry. For antigen retrieval, slides were incubated for 15 min at 95°C in citrate buffer (10 mM, pH 6). The primary antibody was used in a 1:250 dilution for overnight incubation at 4°C. This incubation was followed by a rabbit anti-rat (1:100; P0450; Dako) and a peroxidase-conjugated goat anti-rabbit polyclonal Ab (1:100; P0448; Dako). Peroxidase activity was visualized by the addition of 3,3′-diaminobenzidine. Strong membrane staining above background noise was considered positive and was identified with both the combined and DAB-only view (QuPath 0.1.2). Positive cell identification was determined unblinded. Tumoral F4/80 staining was quantified by counting positive cells in three representative fields containing both epithelial and stromal tumoral tissue and expressed as the average number of cells per mm^2^.

### Statistical Analysis

Statistical analyses were performed using GraphPad Prism 7.02. Unless otherwise stated, data are presented as mean ± SD. Unpaired t-test served to test differences between two groups. *p*-Values ≤0.05 were considered significant.

## Results

### *In Vitro* Characterization of [^89^Zr]Zr-DFO-*N*-suc-CSF1R-mAb

**Figure 1:**
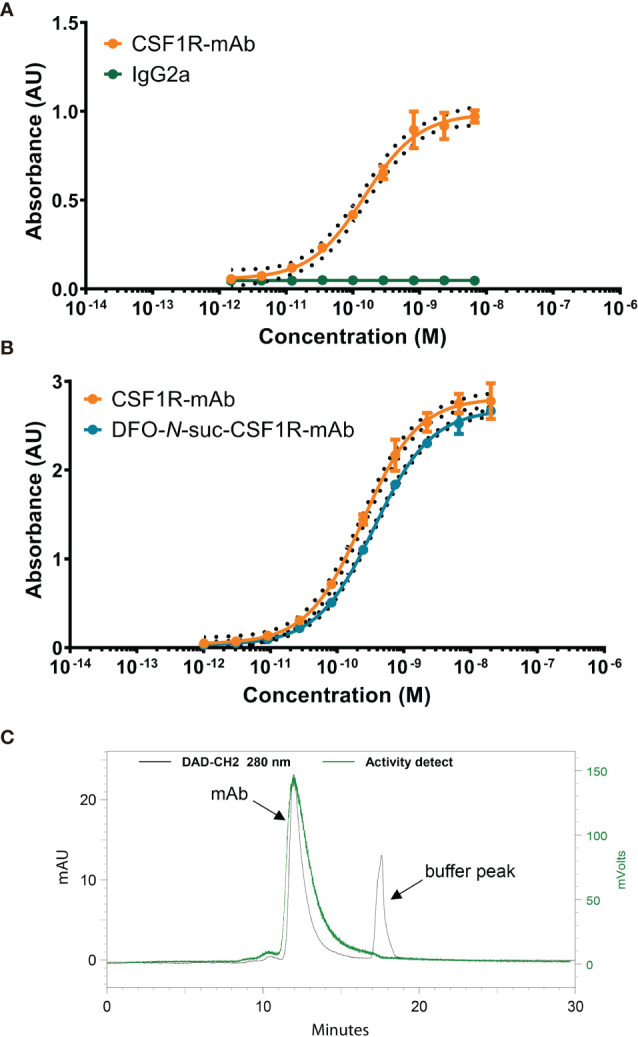
*In vitro* characteristics of CSF1R mAb, DFO-*N*-suc-conjugated, and ^89^Zr-labeled mAb. **(A)** Representative binding curve of CSF1R mAb and IgG2a binding to mouse CSF1R recombinant protein. **(B)** Representative binding curve of DFO-*N*-suc-CSF1R-mAb and CSF1R mAb binding to mouse CSF1R recombinant protein. **(C)** Representative SE HPLC of [^89^Zr]Zr-DFO-*N*-suc-CSF1R-mAb 280-nm signal (black) with the radiochemical signal overlay (green). mAb, monoclonal antibody; AU, absorbance unit; CSF1, colony-stimulating factor 1; CSF1R, colony-stimulating factor 1 receptor; SE HPLC, size-exclusion high-performance liquid chromatography.

CSF1R-mAb binds specifically to CSF1R, while IgG2a does not show binding (**Figure 1A**). The intermediate DFO-N-suc-CSF1R-mAb maintained binding to CSF1R comparable to unconjugated CSF1R-mAb in the ELISA-based binding assay (**Figure 1B**). We successfully radiolabeled CSF1R-mAb and IgG2a ^89^Zr at a specific activity of 60-75 MBq/nmol. Radiochemical purity exceeded 95%, and high molecular weight species were below 5% (**Figure 1C**).

### Biodistribution of 0.4 mg/kg of [^89^Zr]Zr-DFO-*N*-suc-CSF1R-mAb in Non-Tumor-Bearing FVB/N Mice

**Figure 2:**
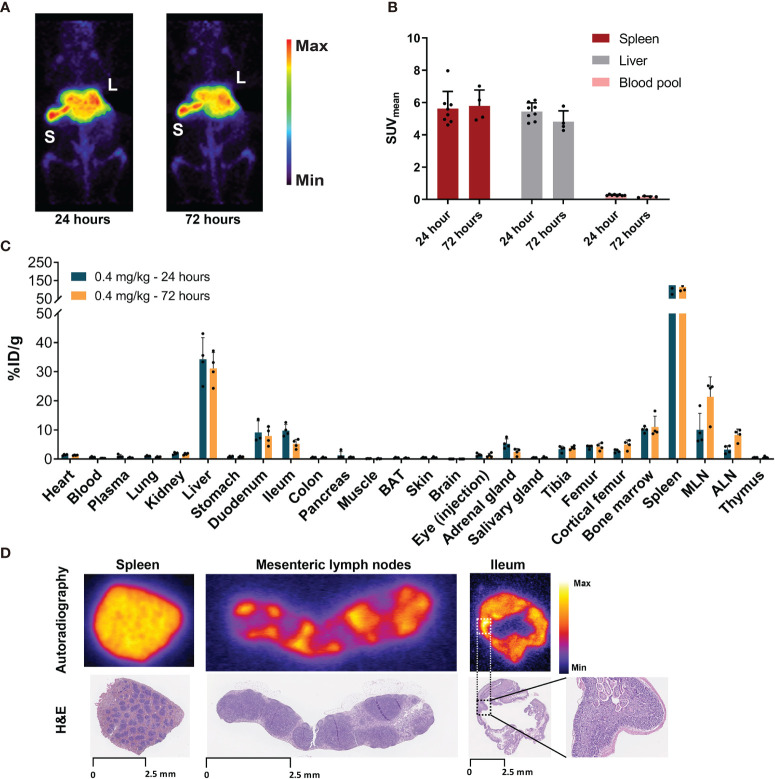
Biodistribution of 0.4 mg/kg of [^89^Zr]Zr-DFO-*N*-suc-CSF1R-mAb in non-tumor-bearing FVB/N mice. **(A)** Representative maximal intensity projection PET images of non-tumor-bearing FVB/N mice 24 and 72 h after intravenous administration of 0.4 mg/kg of [^89^Zr]Zr-DFO-*N*-suc-CSF1R-mAb. **(B)** PET quantification of spleen, liver, and blood pool at 24 (*n* = 8) and 72 (*n* = 4) h after [^89^Zr]Zr-DFO-*N*-suc-CSF1R-mAb administration. Data are represented as mean SUV_mean_ ± SD. **(C)** *Ex vivo* biodistribution at 24 (*n* = 4) and 72 (*n* = 4) h after administration of 0.4 mg/kg of [^89^Zr]Zr-DFO-*N*-suc-CSF1R-mAb intravenously. **(D)** *Ex vivo* autoradiography of spleen, mesenteric lymph node, and ileum tissue (upper panel) and matching H&E staining on the same tissue slide. Spleen, mesenteric lymph node, and ileum were exposed to different phosphor plates. Ileum magnification depicting autolysis. L, liver; S, spleen; BAT, brown adipose tissue; MLN, mesenteric lymph nodes; ALN, axillary lymph nodes; %ID/g, percentage injected dose per gram of tissue.

PET imaging in non-tumor-bearing mice revealed low blood pool levels of [^89^Zr]Zr-DFO-*N*-suc-CSF1R-mAb with SUV_mean_ of 0.3 ± 0.04 at 24 h and 0.2 ± 0.04 at 72 h after injection (**Figures 2A, B**). Spleen uptake showed a mean SUV_mean_ of 5.6 ± 1.1 at 24 h and 5.8 ± 1.0 at 72 h (**Figure 2B**). Similar high uptake was observed in the liver with SUV_mean_ of 5.4 ± 0.5 and 4.8 ± 0.7 at 24 and 72 h, respectively (**Figure 2B**). High uptake in the spleen and liver, with spleen uptake at 72 h after injection of 115 ± 23%ID/g and liver uptake of 31 ± 5%ID/g, was confirmed by *ex vivo* biodistribution (**Figure 2C**). Also, *ex vivo* biodistribution showed uptake in the mesenteric and axillary lymph nodes, duodenum, ileum, and bone marrow (**Figure 2C**). Autoradiography at 72 h showed a ^89^Zr distribution pattern for the spleen overlapping with the macrophage containing red pulp and for the mesenteric lymph nodes overlapping with the macrophage containing non-follicular regions (**Figure 2D**). For the ileum, no specific ^89^Zr distribution pattern was observed, except for some slightly elevated aspecific uptake in regions showing autolysis (**Figure 2D**). Thus, 0.4 mg/kg of [^89^Zr]Zr-DFO-*N*-suc-CSF1R-mAb was distributed quickly to the spleen and liver, with macrophage-specific localization in lymphoid organs.

### Biodistribution of 4 mg/kg of [^89^Zr]Zr-DFO-*N*-suc-CSF1R-mAb in Non-Tumor-Bearing FVB/N Mice

**Figure 3:**
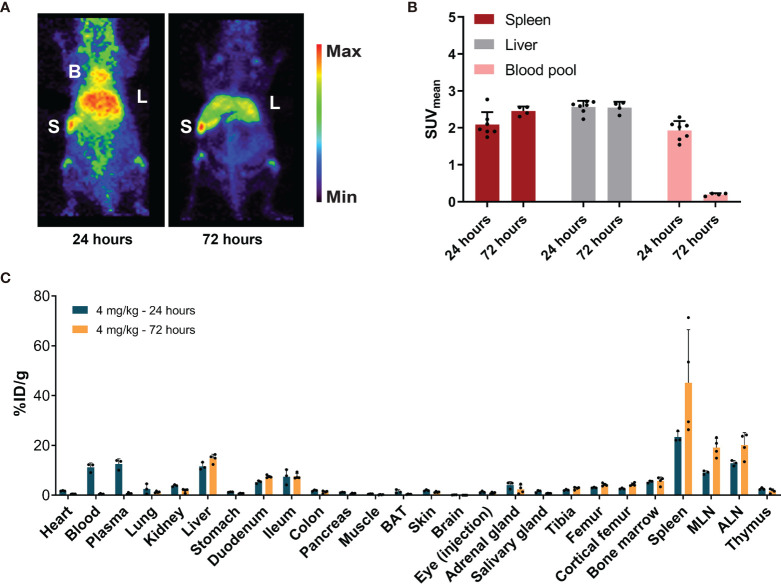
Biodistribution of 4 mg/kg of [^89^Zr]Zr-DFO-*N*-suc-CSF1R-mAb in non-tumor-bearing FVB/N mice. **(A)** Representative maximal intensity projection PET images of non-tumor-bearing FVB/N mice 24 and 72 h after administration of 4 mg/kg of [^89^Zr]Zr-DFO-*N*-suc-CSF1R-mAb intravenously. **(B)** PET quantification of spleen, liver, and blood pool at 24 (*n* = 7) and 72 (*n* = 4) h after [^89^Zr]Zr-DFO-*N*-suc-CSF1R-mAb administration. Data are presented as mean + SD. **(C)** *Ex vivo* biodistribution at 24 (*n* = 4) and 72 (*n* = 4) h after administration of 4 mg/kg of [^89^Zr]Zr-DFO-*N*-suc-CSF1R-mAb intravenously. Data are expressed as mean + SD. B, blood pool; L, liver; S, spleen; SUV_mean_, mean standardized uptake value; BAT, brown adipose tissue; MLN, mesenteric lymph nodes; ALN, axillary lymph nodes; %ID/g, percentage injected dose per gram of tissue.

MicroPET imaging 24 h after 4 mg/kg of [^89^Zr]Zr-DFO-*N*-suc-CSF1R-mAb administration revealed a higher SUV_mean_ of 1.9 ± 0.3 in the blood pool (**Figures 3A, B**) and 11.2 ± 1.8%ID/g by *ex vivo* biodistribution (**Figure 3C**). Again no [^89^Zr]Zr-DFO-*N*-suc-CSF1R-mAb was present in the blood pool 72 h after injection, as shown by PET with a SUV_mean_ of 0.2 ± 0.04 and by *ex vivo* biodistribution 0.4 ± 0.2%ID/g (**Figures 3B, C**). Also for the 4 mg/kg dose after 24 and 72 h, there was clear spleen and liver uptake (**Figure 3A**), but uptake was lower compared with that of the 0.4 mg/kg group at both time points (**Figures 2B** and **3B**). *Ex vivo* biodistribution was in line with microPET findings (**Figure 3C**). *Ex vivo* biodistribution at 24 h after radiolabeled antibody administration further revealed enriched [^89^Zr]Zr-DFO-*N*-suc-CSF1R-mAb in plasma over whole blood levels (**Figure 3C**). In short, this demonstrates that 4 mg/kg of [^89^Zr]Zr-DFO-*N*-suc-CSF1R-mAb marginally increased circulating levels and visualized the spleen and liver.

### Biodistribution of 10 mg/kg of [^89^Zr]Zr-DFO-*N*-suc-CSF1R-mAb in Non-Tumor-Bearing FVB/N Mice

**Figure 4:**
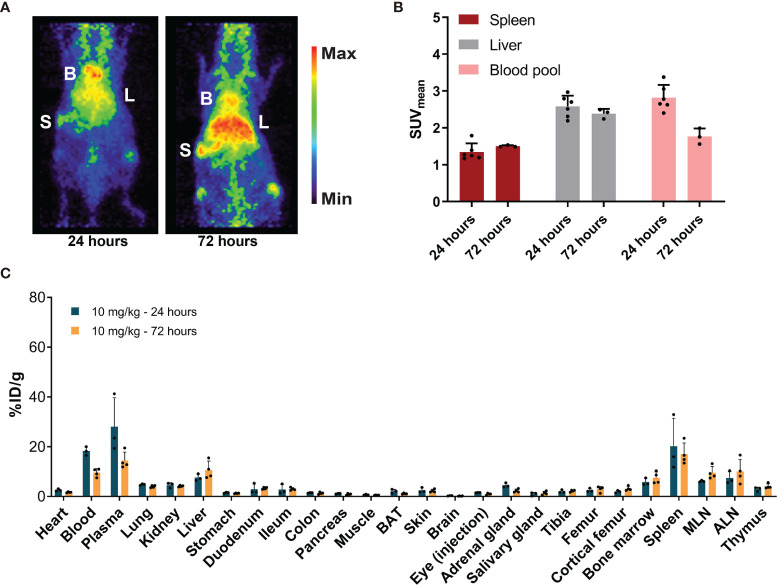
Biodistribution of 10 mg/kg of [^89^Zr]Zr-DFO-*N*-suc-CSF1R-mAb in non-tumor-bearing FVB/N mice. **(A)** Representative maximal intensity projection PET images of non-tumor-bearing FVB/N mice 24 and 72 h after intravenous administration of 10 mg/kg of [^89^Zr]Zr-DFO-*N*-suc-CSF1R-mAb. B, blood pool; L, liver; S, spleen. **(B)** PET quantification of spleen, liver, and blood pool at 24 (*n* = 6) and 72 (*n* = 3) h after [^89^Zr]Zr-DFO-N-suc-CSF1R-mAb administration. Data are presented as mean SUV_mean_ ± SD. **(C)** *Ex vivo* biodistribution at 24 (*n* = 3) and 72 (*n* = 4) h after administration of 10 mg/kg of [^89^Zr]Zr-DFO-*N*-suc-CSF1R-mAb. Data are expressed as mean + SD. B, blood pool; L, liver; S, spleen; SUV_mean_, mean standardized uptake value; BAT, brown adipose tissue; MLN, mesenteric lymph nodes; ALN, axillary lymph nodes; %ID/g, percentage injected dose per gram of tissue.

After administration of 10 mg/kg of [^89^Zr]Zr-DFO-*N*-suc-CSF1R-mAb, microPET visualized blood pool as well as the liver and spleen (**Figure 4A**). Blood pool levels at 24 and 72 h showed a SUV_mean_ of 2.8 ± 0.4 and 1.8 ± 0.2, respectively (**Figure 4B**). Spleen SUV_mean_ was 1.3 ± 0.2 at 24 h and 1.5 ± 0.02 at 72 h after radiolabeled antibody administration (**Figure 4B**). Liver SUV_mean_ was 2.6 ± 0.3 at 24 h and 2.4 ± 0.1 at 72 h. *Ex vivo* biodistribution confirmed PET results, with a high presence in blood pool and high uptake in the liver and spleen 24 and 72 h after radiolabeled antibody administration (**Figure 4C**). *Ex vivo* biodistribution showed for the liver, spleen, duodenum, and ileum no change in uptake between 24 and 72 h after radiolabeled antibody administration (**Figure 4C**).

Comparing all three dose groups, *ex vivo* biodistribution of [^89^Zr]Zr-DFO-*N*-suc-CSF1R-mAb showed a clear dose-dependent increase in blood levels (**Supplementary Figures 1A, B**), with the lowest dose rapidly eliminating from circulation and distributing predominantly to the liver and spleen. Increasing the antibody protein dose decreased uptake in the spleen and liver and increased blood levels of [^89^Zr]Zr-DFO-*N*-suc-CSF1R-mAb at 24 h (**Supplementary Figure 1A**) and 72 h (**Supplementary Figure 1B**). Increasing antibody protein dose trended to a dose-dependent decrease in duodenum and ileum uptake (**Supplementary Figures 1A, B**).

### Uptake of [^89^Zr]Zr-DFO-*N*-suc-CSF1R-mAb and [^89^Zr]Zr-DFO-*N*-suc-IgG2a in KEP Tumor-Bearing FVB/N Mice

**Figure 5:**
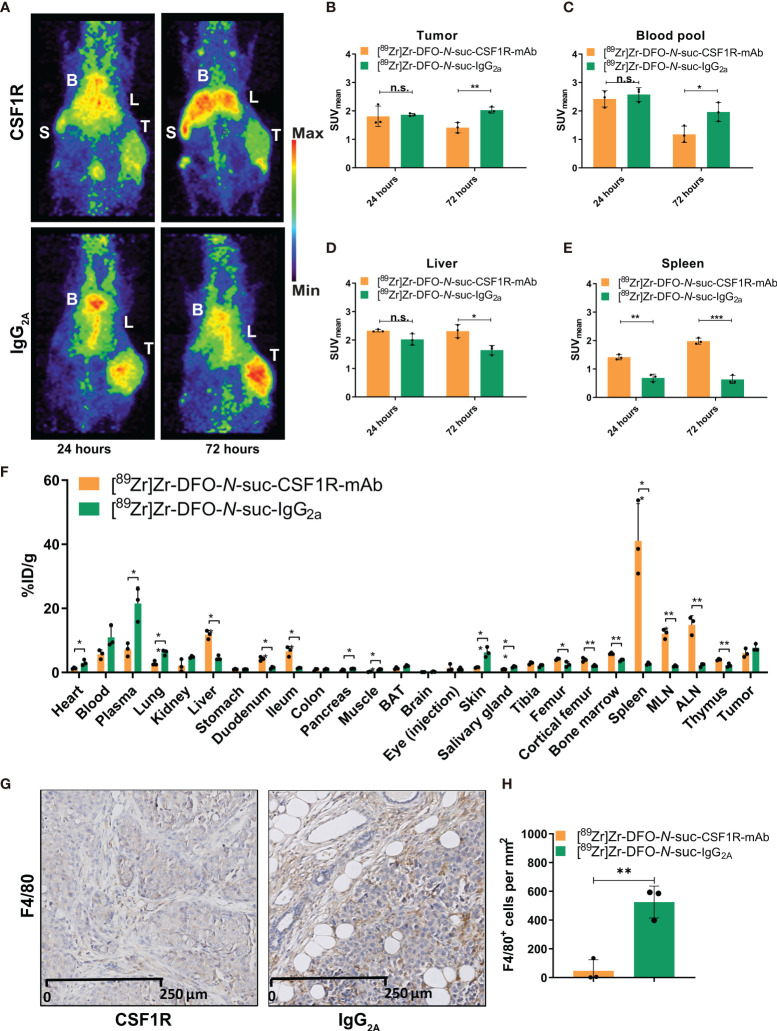
Biodistribution of 10 mg/kg of [^89^Zr]Zr-DFO-*N*-suc-CSF1R-mAb and [^89^Zr]Zr-DFO-*N*-suc-IgG2a antibody in KEP tumor-bearing FVB/N mice. **(A)** Representative maximal intensity projection PET images of KEP tumor-bearing FVB/N mice 24 and 72 h after intravenous administration of 10 mg/kg of [^89^Zr]Zr-DFO-*N*-suc-CSF1R-mAb or [^89^Zr]Zr-DFO-*N*-suc-IgG2a antibody. PET quantification of **(B)** tumor, **(C)** blood pool, **(D)** liver, and **(E)** spleen at 24 (*n* = 3) and 72 (*n* = 3) h after [^89^Zr]Zr-DFO-*N*-suc-CSF1R-mAb or [^89^Zr]Zr-DFO-*N*-suc-IgG2a antibody administration. Data are presented as mean + SD. **(F)** *Ex vivo* biodistribution at 72 h after administration of 10 mg/kg of [^89^Zr]Zr-DFO-*N*-suc-CSF1R-mAb (*n* = 3) or [^89^Zr]Zr-DFO-*N*-suc-IgG2a (*n* = 3) antibody. Data are expressed as mean + SD. **(G)** Representative immunohistochemistry of F4/80 in KEP tumors of FVB/N mice at 72 h after administration of [^89^Zr]Zr-DFO-*N*-suc-CSF1R-mAb or [^89^Zr]Zr-DFO-*N*-suc-IgG2a intravenously. **(H)** Quantification of tumoral F4/80 immunohistochemistry. *p ≤ 0.05; **p ≤ 0.01; ***p ≤ 0.001 (unpaired t-test). B, blood pool; L, liver; S, spleen; T, tumor; SUV_mean_, mean standardized uptake value; BAT, brown adipose tissue; MLN, mesenteric lymph nodes; ALN, axillary lymph nodes; %ID/g, percentage injected dose per gram of tissue. n.s., not significant.

As 10 mg/kg of [^89^Zr]Zr-DFO-*N*-suc-CSF1R-mAb showed blood pool levels up to 72 h, thereby allowing sufficient time for circulating antibody to potentially reach the tumor, we compared the biodistribution of 10 mg/kg of [^89^Zr]Zr-DFO-*N*-suc-CSF1R-mAb and 10 mg/kg of isotype control [^89^Zr]Zr-DFO-*N*-suc-IgG2a in orthotopic KEP tumor-bearing FVB/N mice. The tumor, liver, and heart region, representing the blood pool, showed visual uptake by microPET with both radiolabeled antibodies (**Figure 5A**). The spleen was only visualized following [^89^Zr]Zr-DFO-*N*-suc-CSF1R-mAb administration (**Figure 5A**). At 72 h after [^89^Zr]Zr-DFO-*N*-suc-IgG2a administration, there was a higher presence in tumor and blood pool and less in the liver and spleen than for [^89^Zr]Zr-DFO-*N*-suc-CSF1R-mAb (**Figures 5B–E**). When corrected for blood pool levels, tumor SUV_mean_ was similar for both radiolabeled antibodies (data not shown). This was confirmed by *ex vivo* biodistribution, which also showed no specific tumor uptake of [^89^Zr]Zr-DFO-*N*-suc-CSF1R-mAb (**Figure 5F**). *Ex vivo* analyses showed at 72 h higher uptake of [^89^Zr]Zr-DFO-*N*-suc-CSF1R-mAb than [^89^Zr]Zr-DFO-*N*-suc-IgG2 in primary and secondary lymphoid tissues. These included the spleen, mesenteric lymph nodes, axillary lymph nodes, thymus, and bone marrow (**Figure 5F**). In addition, specific uptake of [^89^Zr]Zr-DFO-*N*-suc-CSF1R-mAb was observed in the liver, duodenum, and ileum (**Figure 5F**). Ten-fold fewer macrophages were observed in tumors from mice that received 10 mg/kg of [^89^Zr]Zr-DFO-*N*-suc-CSF1R-mAb (47 ± 77 per mm^2^) compared with [^89^Zr]Zr-DFO-*N*-suc-IgG2a (525 ± 111 per mm^2^) as assessed by immunohistochemistry (**Figures 5G, H**). Staining of a mesenteric lymph node of a mouse treated with 10 mg/kg of [^89^Zr]Zr-DFO-*N*-suc-CSF1R-mAb without primary antibody showed no signal, indicating no interference of the CSF1R mAb with immunohistochemical staining (**Supplementary Figure 2**).

## Discussion

This is the first molecular imaging study to analyze the biodistribution of a CSF1R mAb. A low protein dose of [^89^Zr]Zr-DFO-*N*-suc-CSF1R-mAb resulted within 24 h in antibody elimination from the blood pool due to distribution to CSF1R-rich organs, such as the liver, spleen, lymph nodes, duodenum, and ileum. Increasing the protein dose up to 10 mg/kg resulted in circulating antibody levels up to 72 h. There was CSF1R-specific uptake by macrophages in the spleen and liver but not in the tumor with [^89^Zr]Zr-DFO-*N*-suc-CSF1R-mAb most likely due to antibody-mediated depletion of intratumoral macrophages.

Macrophages are widely spread across many organs in which they are involved in tissue homeostasis. Many different tissue-resident macrophages express CSF1R, such as Kupffer cells in the liver, red pulp macrophages in the spleen, and macrophages in the intestine (25–27). Besides, macrophages can have tumor-promoting characteristics in the tumor microenvironment (5–12). The CSF1R mAb has to reach the tumor to deplete these protumor macrophages. This study demonstrates that [^89^Zr]Zr-DFO-*N*-suc-CSF1R-mAb is not exclusively targeting tumor macrophages but preferably distributes to other organs with high macrophage presence such as the liver and spleen, removing the antibody from circulation. Due to the therapeutic effects of the high dose required to establish circulating antibody, imaging macrophages in other organs seems not possible with this approach. The low number of intratumoral macrophages observed in our study after 10 mg/kg of [^89^Zr]Zr-DFO-*N*-suc-CSF1R-mAb administration can explain the lack of specific tumor uptake of [^89^Zr]Zr-DFO-*N*-suc-CSF1R-mAb. [^89^Zr]Zr-DFO-*N*-suc-CSF1R-mAb still reached the tumor, and due to the relatively high protein dose, CSF1R-positive macrophages were already eliminated within the 72-h exposure. Although macrophage depletion at 24 h after antibody administration could not be assessed in this study, future studies with macrophage-targeting agents might want to address macrophage depletion or reprogramming at multiple time points during treatment course. This might help to better understand their *in vivo* behavior and streamline drug development. CSF1R single antibody activity on the tumor microenvironment in this tumor model was also reported earlier (11). In this study, 225-mm^3^ KEP tumors were treated with 60 mg/kg of CSF1R mAb intraperitoneally loading dose and 30 mg/kg intraperitoneally once per week, corresponding to 1.5 and 0.75 mg based on a 25-g mouse (11). CSF1R mAb-treated tumors showed less tumoral macrophages as compared with control treatment as assessed by immunohistochemistry and flow cytometry (11). In that study, CSF1R mAb alone, however, did not demonstrate antitumor effects, whereas the combination with cisplatin showed synergistic antitumor effects (11).

Similar to our study, using single-photon emission CT (SPECT) isotope indium-111 (^111^In)-labeled antibody targeting the pan-mouse macrophage marker F4/80, antibody tumor uptake did not differ from isotype control in a human breast cancer cell line MDA-MD-231 xenograft (28). When corrected for blood pool levels, tumor uptake was higher for ^111^In-labeled anti-F4/80 than isotype control. However, this was only tested at a low protein dose of 10 µg (~0.4 mg/kg) and, thus, a major difference in elimination half-life. This tracer was studied in an immunodeficient severe combined immunodeficiency (SCID)/beige mouse model with an impaired immune system to allow the engraftment of a human breast cancer xenograft. The impaired immune system and a xenograft tumor make it challenging to translate these results into an immunocompetent model. Noteworthy, F4/80 has no human macrophage equivalent and is therefore not a drug target. Another SPECT study in mice used a radiolabeled antibody against a different macrophage marker, namely, CD206. In that study, biodistribution was determined as early at 24 h after ^125^I-labeled tracer administration reporting whole blood pool levels of 10%ID/g (29). Of interest, in that study, specific tumor uptake was observed. We did not use a CD206-mAb, as CD206 showed low expression by the TAMs in our model (11).

By *ex vivo* biodistribution in our study, high specific uptake was observed in the duodenum and ileum. This uptake could be explained by the presence of an abundant number of macrophages in the lamina propria of the murine small intestine (26). PET allowed us to demonstrate the uptake in the liver, spleen, and blood over time. Nevertheless, PET was unable to detect clear uptake in lymph nodes and the intestine, possibly related to the detection limit of the camera. Gastrointestinal specificity is in line with specific duodenum uptake observed with *ex vivo* biodistribution in a study using ^111^In-labeled F4/80 mAb (28).

In our study at a dose of 0.4 mg/kg, low blood pool levels of CSF1R mAb were observed at 24 h post-administration. The extensive availability of the CSF1R target as a macrophage marker in organs such as the liver and spleen might act as an antibody sink. Besides CSF1R-mediated clearance to the liver and spleen, it cannot be excluded that interaction between the IgG_2a_ backbone of the CSF1R mAb and mouse Fc-receptor partially impacted distribution to the spleen and liver at lower antibody dose levels. For future studies, distribution of IgG_2a_ control at all dose levels might be considered. Similarly to our study, ^111^In-labeled F4/80-mAb demonstrated low blood pool levels and high uptake in the liver and spleen at 24 h post-injection of a 10-µg tracer (28). In that study, increasing the protein dose by 10-fold only slightly increased blood pool levels of ^111^In-F4/80-mAb. Besides, SPECT visualized the kidneys at 24 h post 100-µg tracer administration, which questions the tracer’s *in vivo* stability.

Similar to our findings, fast serum clearance of free CSF1 mAb was observed in a clinical trial with pharmacokinetic analysis of healthy volunteers of a clinical mAb binding CSF1. This trial suggested target-mediated drug disposition as the mechanism responsible for the decline of free CSF1 mAb in serum at low doses up to 5 mg/kg (30). In oncology, many CSF1/CSF1R-targeting drugs are in clinical trials, often in combination with immune checkpoint blockade (1). In early-phase clinical trials in patients with advanced cancer, elevation of liver enzymes has been observed with the CSF1 mAb AMG 820 and the CSF1R mAb MCS110 (31, 32). This increase could be related to the depletion of CSF1R-positive Kupffer cells without any actual liver tissue damage, as seen in cynomolgus macaques treated with a CSF1 mAb (33). In turn, this could be linked to our observation of high liver uptake of [^89^Zr]Zr-DFO-*N*-suc-CSF1R-mAb. The observed decrease in liver uptake upon higher CSF1R-mAb dose could be a result of antigen saturation at the target site, the depletion of Kupffer cells in the liver, or a combination.

As targeting the CSF1–CSF1R axis can result in a potential pan-macrophage depletion, reprogramming or activating TAMs more specifically to a more antitumor role may elicit additional benefits. An example is targeting the macrophage receptor with collagenous structure (MARCO). This scavenging receptor is constitutively expressed by subpopulations of macrophages, particularly those of the spleen’s marginal zone and lymph node medulla and by residential peritoneal macrophages (34). MARCO expression on liver macrophages showed conflicting results, with both absence and higher immunohistochemical MARCO expression on peritumoral macrophages compared with intratumoral macrophages of hepatocellular carcinoma (34–36). MARCO is expressed by TAMs in human breast cancer and correlated with poor clinical outcome (37, 38). Anti-MARCO mAb arrested tumor growth and lowered metastasis in a mouse 4T1 mammary carcinoma model by reprogramming TAMs (36). No clinical trials for MARCO targeting therapy are described. Biodistribution of such novel therapeutics is still unknown, and future studies may provide more insight in TAM biology and support the development of drugs selectively targeting TAMs. In addition, treatment with anti-MARCO mAb could be followed up by imaging macrophage surface markers to study pharmacodynamics.

Our study highlights the need for more biodistribution studies to enhance our understanding of macrophage-targeting antibodies. These studies likely need to address dose, timing, antibody format, and physiological expression of the target to get a comprehensive overview of the pharmacokinetic and pharmacodynamic properties of macrophage-targeting drug. Once such features are identified in the preclinical setting, clinical imaging studies should consider these in their trial design. Studying CSF1R-targeting antibody biodistribution in humans may support elucidating the role of CSF1R-positive macrophages in healthy tissues as well as breast cancer treatment and optimizing (combination) targeting strategies.

## Data Availability Statement

The original contributions presented in the study are included in the article/**Supplementary Material**. Further inquiries can be directed to the corresponding author.

## Ethics Statement

The animal study was reviewed and approved by Institutional Animal Care and Use Committee of the University of Groningen and the Netherlands Cancer Institute.

## Author Contributions

Study concept and design: SW, KV, and CS. Acquisition of data: SW, FS, CH, and KV. Analysis and interpretation of data: all authors. Study supervision: KV, CS, ML, and EV. Writing, review, and revision of the manuscript: all authors. All authors contributed to the article and approved the submitted version.

## Conflict of Interest

EV reports institutional financial support for her advisory role from Daiichi Sankyo, Merck, NSABP, Pfizer, Sanofi, and Synthon and institutional financial support for clinical trials or contracted research from Amgen, AstraZeneca, Bayer, Chugai Pharma, CytomX Therapeutics, G1 Therapeutics, Genentech, Nordic Nanovector, Radius Health, Roche, Synthon, and Servier. CS reports receiving unrestricted research grants from Novartis, Roche, Genentech, Pfizer, SNS Oncology, and G1 Therapeutics that were made available to UMCG. KdV reports receiving research grants from Roche and is a consultant for Third Rock Ventures.

The remaining authors declare that the research was conducted in the absence of any commercial or financial relationships that could be construed as a potential conflict of interest.

## Publisher’s Note

All claims expressed in this article are solely those of the authors and do not necessarily represent those of their affiliated organizations, or those of the publisher, the editors and the reviewers. Any product that may be evaluated in this article, or claim that may be made by its manufacturer, is not guaranteed or endorsed by the publisher.
