# Supplementary material for: Radiolabeled Monoclonal Antibody Against Colony-Stimulating Factor 1 Receptor Specifically Distributes to the Spleen and Liver in Immunocompetent Mice
Source: Front Oncol. 2021 Dec 16;11:786191. doi: 10.3389/fonc.2021.786191 (PMC8716378; doi:10.3389/fonc.2021.786191)
Supplement: Supplementary file 1 [file DataSheet_1.docx]

Supplementary Material

**Supplementary Figure**

**
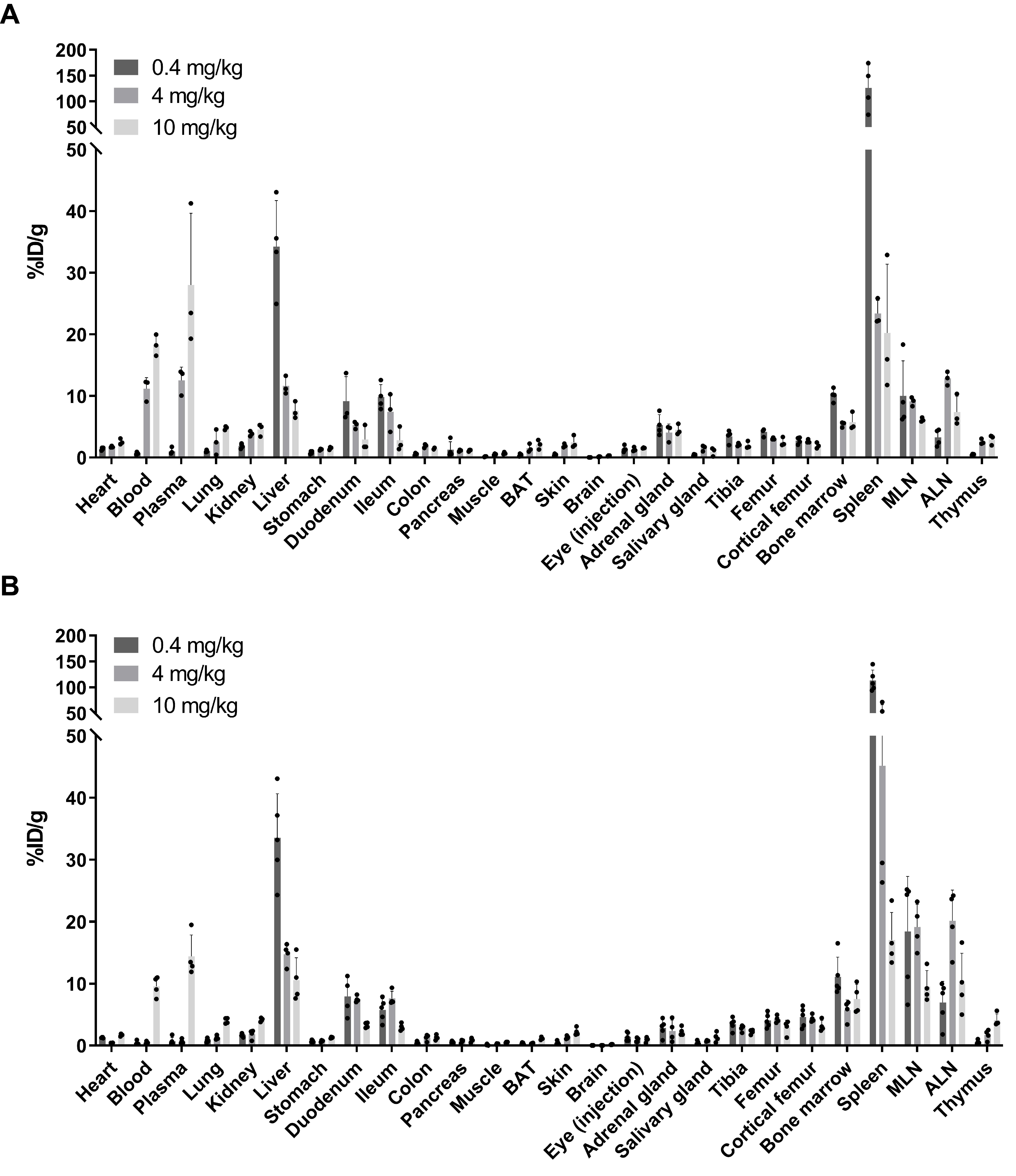
**

**Supplemental Figure 1**. *Ex vivo* biodistribution of 0.4, 4 and 10 mg/kg [^89^Zr]Zr-DFO-*N* suc-CSF1R-mAb in non-tumor-bearing FVB/N mice. **(A)** *Ex vivo* biodistribution at 24 hours after administration of 0.4 (*n* = 4), 4 (*n* = 4) or 10 mg/kg (*n* = 3) [^89^Zr]Zr-DFO-*N*-suc-CSF1R-mAb intravenously. Data are expressed mean + standard deviation. **(B)** *Ex vivo* biodistribution at 72 hours after administration of 0.4 (*n* = 4), 4 (*n* = 4) or 10 mg/kg (*n* = 4) [^89^Zr]Zr-DFO-*N*-suc-CSF1R-mAb. Data are expressed as mean + standard deviation. BAT, brown adipose tissue; MLN, mesenteric lymph nodes; ALN, axillary lymph nodes. % ID/g, percentage injected dose per gram of tissue.


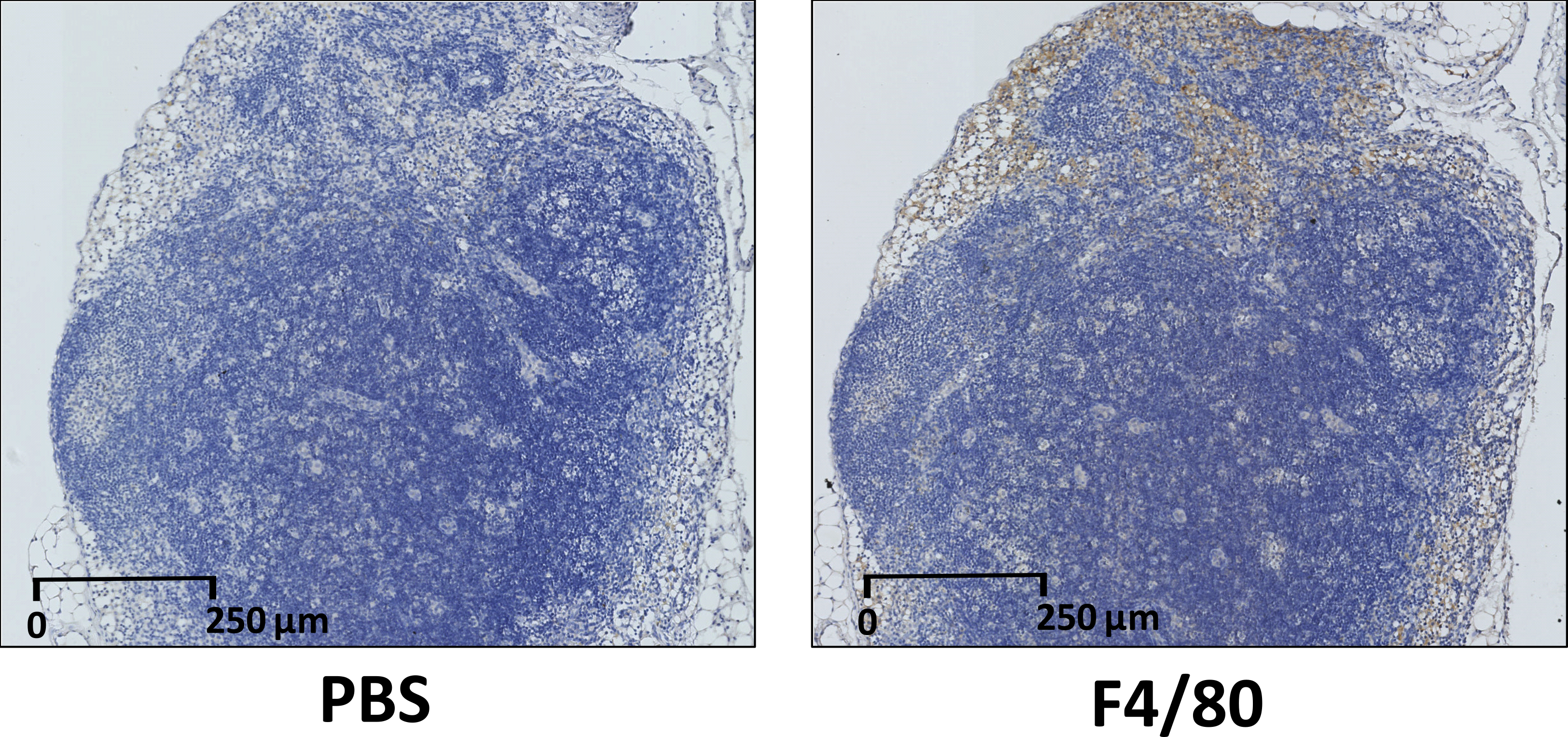


**Supplemental Figure 2.** Immunohistochemical analysis of a mesenteric lymph node from a mouse treated with 10 mg/kg [^89^Zr]Zr-DFO-*N*-suc-CSF1R-mAb at 72 hours after intravenous administration. stained with PBS or F4/80 antibody.
